# Association Between Donor-Recipient Biological Relationship and Allograft Outcomes After Living Donor Kidney Transplant

**DOI:** 10.1001/jamanetworkopen.2021.5718

**Published:** 2021-04-13

**Authors:** S. Ali Husain, Kristen L. King, Navin Sanichar, R. John Crew, Jesse D. Schold, Sumit Mohan

**Affiliations:** 1 Department of Medicine, Division of Nephrology, Columbia University Medical Center, New York, New York; 2 The Columbia University Renal Epidemiology (CURE) Group, New York, New York; 3 Department of Biostatistics, Mailman School of Public Health, Columbia University, New York, New York; 4 Center for Populations Health Research, Lerner Research Institute, Cleveland Clinic, Cleveland, Ohio; 5 Department of Quantitative Health Sciences, Cleveland Clinic, Cleveland, Ohio; 6 Department of Epidemiology, Mailman School of Public Health, Columbia University, New York, New York

## Abstract

**Importance:**

The proportion of living donor kidney transplants from donors unrelated to their recipients is increasing in the US.

**Objective:**

To examine the association between donor-recipient biological relationship and allograft survival after living donor kidney transplant.

**Design, Setting, and Participants:**

This retrospective cohort study used Organ Procurement and Transplantation Network data on US adult living donor kidney transplants (n = 86 154) performed from January 1, 2000, to December 31, 2014, excluding cases in which recipients previously received a kidney transplant (n = 10 342) or key data were missing (n = 2832). Last follow-up was March 20, 2020.

**Exposures:**

Donor-recipient biological relationship.

**Main Outcomes and Measures:**

The primary outcome was death-censored allograft failure. Univariate and multivariable time-to-event analyses were performed for death-censored allograft failure for the overall cohort, then separately for recipients with and without primary diagnoses of cystic kidney disease and for transplants from African American and non–African American donors.

**Results:**

Among the 72 980 transplant donor and recipients included in the study (median donor age, 41 years; interquartile range [IQR], 32-50 years; 43 990 [60%] female; 50 014 [69%] White), 43 174 (59%) donors and recipients were biologically related and 29 806 (41%) were unrelated. Donors related to their recipients were younger (median [IQR] age, 39 [31-48] vs 44 [35-52] years) and less likely to be female (24 848 [58%] vs 19 142 [64%]) or White (26 933 [62%] vs 23 081 [77%]). Recipients related to their donors were younger (median [IQR] age, 48 [34-58] vs 50 [40-58] years), more likely to be female (18 035 [42%] vs 10 530 [35%]), and less likely to have cystic kidney disease (2530 [6%] vs 4600 [15%]). Related pairs had fewer HLA mismatches overall (median [IQR], 3 [2-3] vs 5 [4-5]). After adjustment for HLA mismatches, donor and recipient characteristics, and transplant era, donor-recipient biological relationship was associated with higher death-censored allograft failure (hazard ratio, 1.05; 95% CI, 1.01-1.10; *P* = .03). When stratified by primary disease, this association persisted only for recipients without cystic kidney disease. When stratified by donor race, this association persisted only for transplants from African American donors.

**Conclusions and Relevance:**

In this cohort study, living donor kidney transplants from donors biologically related to their recipients had higher rates of allograft failure than transplants from donors unrelated to their recipients after HLA matching was accounted for. Further study is needed to determine which genetic or socioenvironmental factors are associated with this finding.

## Key Points

**Question:**

What is the association between donor-recipient biological relationship and outcomes after living donor kidney transplantation?

**Findings:**

In this cohort study including more than 70 000 transplants, after taking donor, recipient, and immunologic factors into account, transplants from donors who were biologically related to their recipients had a higher rate of allograft failure compared with transplants from unrelated donors.

**Meaning:**

These findings suggest that kidney donors who are related to their recipients may share genetic or socioenvironmental predispositions to kidney disease that shorten allograft longevity.

## Teaser

This cohort study uses US adult living donor kidney transplant data to examine the association between donor-recipient biological relationship and allograft survival after living donor kidney transplant.

## Introduction

Despite efforts to increase kidney transplant rates in the US, the annual number of living kidney donors has stagnated during the past 2 decades.^1^ As a result, the proportion of transplants from living donors has decreased, now accounting for fewer than one-third of domestic kidney transplants.^1^ At the same time, the characteristics of living donors are also changing. Among the most notable shifts is a marked increase in the frequency of living kidney donation from donors without biological relationships to their recipients. In 2000, approximately 1 in 4 donor-recipient pairs was not biologically related; by 2019, this proportion had more than doubled.^1^

The association between the marked increase in living unrelated kidney transplant and allograft outcomes is incompletely understood. Prior investigators^2,3,4,5,6,7,8^ found similar allograft survival for recipients who are related to their donors and those who are not, although these analyses have not consistently accounted for the association between biological relationship and HLA mismatching. Of importance, unrelated recipients have a higher likelihood of acute rejection early after transplant, perhaps reflecting a greater degree of HLA matching when donors and recipients are related.^6,7,8,9^ However, related donor-recipient pairs may also share genetic predispositions to kidney disease and/or decreased kidney function, potentially partly explaining the higher rate of postdonation kidney failure observed in living donors who are related to their recipients.^10,11,12^ Given that some transplant candidates may have multiple potential donors from whom to choose, a better understanding of the association between donor-recipient biological relationship and posttransplant outcomes can help inform donor selection. We therefore sought to determine whether living donor kidney transplants from biologically related donors have higher rates of allograft failure after accounting for degree of HLA mismatching.

## Methods

We identified all adult donor-recipient pairs for living donor kidney transplants performed in the US from January 1, 2000, to December 31, 2014, using the Organ Procurement and Transplantation Network Standard Transplant Analysis and Research file (2020Q1). This study interval was selected to ensure at least 5 years of follow-up for all transplant recipients. After excluding prior transplant recipients and pairs missing data on key variables, a final cohort for the primary analysis comprised 72 980 donor-recipient pairs (eFigure 1 in the Supplement). Patient-level data in this registry are deidentified. Informed consent was not required because of the use of deidentified data. The study was approved by the institutional review board of Columbia University Medical Center. All research activities associated with this study were consistent with the principles of the Declaration of Istanbul.^13^ This study followed the Strengthening the Reporting of Observational Studies in Epidemiology (STROBE) reporting guideline.

Donor-recipient pairs were classified as related if any biological relationship was reported (eMethods in the Supplement). Number of HLA mismatches (0, 1, or 2) were counted for each locus HLA-A, -B, and -DR. Classification of other variables is detailed in the eMethods in the Supplement. Last follow-up was March 20, 2020.

### Main and Secondary Outcomes

Our primary outcome of interest was death-censored allograft failure (allograft failure censored for death with a functioning allograft). Secondary outcomes were composite allograft failure (death or allograft failure), recipient death, and 1-year rejection.

### Statistical Analysis

Donor and recipient demographic variables and number of HLA matches were compared for related vs unrelated donor-recipient pairs using χ^2^ and Wilcoxon rank sum tests for categorial and continuous variables, respectively. Unadjusted death-censored allograft survival was assessed using the Kaplan-Meier method and log-rank test. We performed univariate and multivariable time-to-event analyses for death-censored allograft failure using Cox proportional hazards regression models for the overall cohort, then separately for recipients with and without a primary diagnosis of cystic kidney disease, and finally for transplants from African American donors and non–African American donors. Model covariates were chosen based on clinical significance. In the first adjusted model, only donor-recipient biological relationship and HLA-A, -B, and -DR mismatches were included. A second model added donor and recipient characteristics, and a third model also added transplant era. Cox proportional hazard regression models were then recalculated using composite allograft failure and recipient death as outcomes.

We next performed post hoc analyses comparing 1-year rejection and causes of allograft failure for related vs unrelated transplants among the subsets of patients with data available for each. The proportion of recipients in each group experiencing rejection within 1 year of transplant was compared using χ^2^ tests, followed by univariate and multivariable logistic regression. Causes of allograft failure for related vs unrelated transplants were compared using χ^2^ tests.

Finally, to determine whether results would be similar if we included transplants that occurred more recently (ie, those with shorter follow-up), we repeated all time-to-event analyses mentioned above using a cohort of transplants performed from January 1, 2000, to December 31, 2019.

Statistical significance was identified by a 2-sided α = .05. All analyses were performed using Stata/MP15 statistical software (StataCorp).

## Results

A total of 72 980 living donor kidney transplants (median donor age, 41 years; interquartile range [IQR], 32-50 years; 43 990 [60%] female; 50 014 [69%] White) that occurred from 2000 to 2014 were included in the primary analytic cohort (eFigure 1 in the Supplement). Donors and recipients were biologically related in 43 174 (59%) and not biologically related in 29 806 transplants (41%) (Table 1). Although the overall number of living donor kidney transplants remained similar throughout the study period’s eras, the proportion of donors not biologically related to their recipients increased from 32% in 2000-2004 to 50% in 2010-2014 (eFigure 2 in the Supplement).

**Table 1.** Baseline Characteristics of Living Donor Kidney Transplant Donors and Recipients Included in the Analysis^a^

Characteristic	All (N = 72 980)	Not related (n = 29 806)	Related (n = 43 174)	*P* value
**Donor characteristics**				
Age, median (IQR), y	41 (32-50)	44 (35-52)	39 (31-48)	<.001
Female sex	43 990 (60)	19 142 (64)	24 848 (58)	<.001
Race				
White	50 014 (69)	23 081 (77)	26 933 (62)	<.001
Black/African American	9499 (13)	2541 (9)	6958 (16)	
Hispanic/Latino	9836 (13)	2967 (10)	6869 (16)	
Predonation serum creatinine, median (IQR), mg/dL	0.8 (0.7-1.0)	0.8 (0.7-1.0)	0.9 (0.7-1.0)	<.001
**Recipient characteristics**				
Age, median (IQR), y	49 (37-58)	50 (40-58)	48 (34-58)	<.001
Female sex	28 565 (39)	10 530 (35)	18 035 (42)	<.001
Race				
White	48 032 (66)	21 111 (71)	26 921 (62)	<.001
Black/African American	10 764 (15)	3731 (13)	7033 (16)	
Hispanic/Latino	9931 (14)	3172 (11)	6759 (16)	
Cause of kidney disease				
Diabetes	13 146 (18)	5766 (19)	7380 (17)	<.001
Hypertension	10 108 (14)	3995 (13)	6113 (14)	
Glomerular disease	16 266 (22)	6557 (22)	9709 (22)	
Cystic kidney disease	7130 (10)	4600 (15)	2530 (6)	
Other or unknown	26 330 (36)	8888 (30)	17 442 (40)	
Panel reactive antibody ≥80%	1520 (2)	601 (2)	919 (2)	.30
Diabetes	20 925 (29)	8660 (29)	12 265 (28)	.06
Peripheral vascular disease	2929 (4)	1230 (4)	1699 (4)	.20
Functional impairment	39 156 (54)	16 990 (57)	22 166 (51)	<.001
Pretransplant dialysis				
Preemptive (no dialysis)	22 572 (31)	9639 (32)	12 933 (30)	<.001
<1 y	8433 (12)	3221 (11)	5212 (12)	
≥1 y	20 389 (28)	8750 (29)	11 639 (27)	
Not preemptive but time not known	21 586 (30)	8196 (28)	13 390 (31)	
Employment status				
Employed	23 078 (32)	11 138 (37)	11 940 (28)	<.001
Not employed	25 261 (35)	10 406 (35)	14 855 (34)	
Unknown or missing	24 641 (34)	8262 (28)	16 379 (38)	
Insurance				
Private	42 967 (59)	18 565 (63)	24 311 (56)	<.001
Medicare	19 373 (27)	7984 (27)	11 389 (26)	
Medicaid	2787 (4)	780 (3)	2007 (5)	
Other	7853 (11)	2386 (8)	5467 (13)	
**Other characteristics**				
ABO-incompatible transplant	883 (1)	452 (2)	431 (1)	<.001
Transplant era				
2000-2004	23 655 (32)	7577 (25)	16 078 (37)	<.001
2005-2009	25 460 (35)	10 342 (35)	15 118 (35)	
2010-2014	23 865 (33)	11 887 (40)	11 978 (28)	

Abbreviation: IQR, interquartile range.

^a^ Data are presented as number (percentage) of patients unless otherwise indicated.

Related donors, compared with unrelated donors, were younger (median [IQR] age, 39 [31-48] vs 44 [35-52] years), less likely to be female (24 848 [58%] vs 19 142 [64%]), and less likely to be White (26 933 [62%] vs 23 081 [77%]) (Table 1). Recipients who were related to their donors were also younger (median [IQR] age, 48 [34-58] vs 50 [40-58] years) and more likely to be female (18 035 [42%] vs 10 530 [35%]) than recipients unrelated to their donors. Recipients of living related transplants were less likely to carry a primary diagnosis of cystic kidney disease than recipients unrelated to their donors (2530 [6%] vs 4600 [15%]). Recipients of related transplants were also less likely to be employed (11 940 [28%] vs 11 138 [37%]) or have private health insurance (24 311 [56%] vs 18 565 [63%]) (Table 1).

Related donor-recipient pairs had higher degrees of HLA matching compared to unrelated pairs (Table 2). Overall, related pairs had a median of 3 mismatches (IQR, 2-3) compared with a median of 5 mismatches (IQR, 4-5) for unrelated pairs. Furthermore, related pairs were much more likely to have 0 HLA mismatches (129 [14%] vs 5935 [0.4%]) and much less likely to have 6 HLA mismatches (1506 [3%] vs 5782 [19%]) compared with unrelated pairs. These differences were also observed when examining the number (IQR) of HLA-A (1 [0-1] vs 1 [1-2]), HLA-B (1 [0-1] vs 2 [1-2]), and HLA-DR (1 [0-1] vs 1 [1-2]) mismatches individually (Table 2). A greater degree of HLA mismatching was associated with increased risk of allograft failure (eFigure 3 in the Supplement).

**Table 2.** HLA Mismatches in Biologically Related vs Unrelated Living Kidney Donor-Recipient Pairs

HLA type	No. (%) of donor-recipient pairs			*P* value
	All (N = 72 980)	Not related (n = 29 806)	Related (n = 43 174)	
Total				
0	6064 (8)	129 (0.4)	5935 (14)	<.001
1	3824 (5)	271 (0.9)	3553 (8)	
2	11 821 (16)	1253 (4)	10 568 (24)	
3	19 928 (27)	4151 (14)	15 777 (37)	
4	11 025 (15)	8108 (27)	2917 (7)	
5	13 030 (18)	10 112 (34)	2918 (7)	
6	7288 (10)	5782 (19)	1506 (3)	
HLA-A				
0	16 463 (23)	2651 (9)	13 812 (32)	<.001
1	38 494 (53)	13 204 (44)	25 290 (59)	
2	18 023 (25)	13 951 (47)	4072 (9)	
HLA-B				
0	11 169 (15)	940 (3)	10 229 (24)	<.001
1	36 160 (50)	9043 (30)	27 117 (63)	
2	25 651 (35)	19 823 (67)	5828 (14)	
HLA-DR				
0	14 661 (20)	1988 (7)	12 673 (29)	<.001
1	38 412 (53)	12 517 (42)	25 895 (60)	
2	19 907 (27)	15 301 (51)	4606 (11)	

Allograft failure was observed after 5214 (17%) unrelated and 8155 (19%) related transplants, whereas recipient death was observed after 5162 (17%) unrelated and 8205 (19%) related transplants. Death-censored allograft failure was similar after related and unrelated transplants (eFigure 4 in the Supplement). However, unrelated pairs had longer death-censored allograft survival at any given number of HLA mismatches greater than 1 (eFigure 5 in the Supplement). After adjustment for the number of HLA-A, -B, and -DR mismatches, donor-recipient biological relationship was associated with a 26% increased rate of death-censored allograft failure (hazard ratio [HR], 1.26; 95% CI, 1.21-1.31; *P* < .001) (Table 3). After further adjustment for donor and recipient characteristics, kidney transplants from related donors remained associated with increased death-censored allograft failure (HR, 1.06; 95% CI, 1.02-1.11; *P* = .005). Finally, further inclusion of study era attenuated the association, which remained significant (HR, 1.05; 95% CI, 1.01-1.10; *P* = .03) (Table 3).

**Table 3.** Association Between Donor-Recipient Biological Relationship and Outcomes After Living Donor Kidney Transplant^a^

	Unadjusted		Model 1		Model 2		Model 3	
	HR (95% CI)	*P* value	HR (95% CI)	*P* value	HR (95% CI)	*P* value	HR (95% CI)	*P* value
**Death-censored graft failure**								
Donor related to recipient, full cohort	1.01 (0.97-1.04)	.77	1.26 (1.21-1.31)	<.001	1.06 (1.02-1.11)	.005	1.05 (1.01-1.10)	.03
Donor related to recipient, cystic kidney disease^b^	0.91 (0.78-1.07)	.26	1.08 (0.90-1.30)	.40	1.04 (0.86-1.25)	.68	1.03 (0.85-1.24)	.77
Donor related to recipient, noncystic kidney disease^c^	0.95 (0.91-0.98)	.004	1.2 (1.15-1.25)	<.001	1.07 (1.02-1.11)	.005	1.05 (1.01-1.10)	.03
Donor related to recipient, donor African American^d^	1.03 (0.95-1.12)	.49	1.19 (1.07-1.31)	.001	1.13 (1.02-1.25)	.02	1.12 (1.01-1.23)	.03
Donor related to recipient, donor not African American^e^	0.93 (0.90-0.97)	<.001	1.15 (1.10-1.21)	<.001	1.04 (1.00-1.10)	.08	1.03 (0.98-1.08)	.20
**Composite graft failure**								
Donor related to recipient, full cohort	1.02 (0.99-1.04)	.25	1.16 (1.13-1.20)	<.001	1.13 (1.09-1.17)	<.001	1.12 (1.08-1.15)	<.001
Donor related to recipient, cystic kidney disease^b^	0.96 (0.86-1.07)	.46	1.06 (0.93-1.20)	.42	1.06 (0.93-1.22)	.37	1.06 (0.92-1.21)	.43
Donor related to recipient, non–cystic kidney disease^c^	0.96 (0.93-0.99)	.002	1.11 (1.07-1.14)	<.001	1.13 (1.10-1.17)	<.001	1.12 (1.08-1.16)	<.001
Donor related to recipient, donor African American^d^	1.05 (0.98-1.13)	.17	1.17 (1.07-1.27)	<.001	1.13 (1.03-1.22)	.006	1.11 (1.02-1.21)	.02
Donor related to recipient, donor not African American^e^	0.98 (0.95-1.00)	.09	1.11 (1.07-1.15)	<.001	1.11 (1.07-1.15)	<.001	1.10 (1.06-1.14)	<.001
**Recipient death**								
Donor related to recipient, full cohort	1.00 (0.96-1.03)	.88	1.05 (1.01-1.10)	.02	1.07 (1.02-1.12)	.003	1.06 (1.01-1.11)	.02
Donor related to recipient, cystic kidney disease^b^	0.95 (0.82-1.11)	.54	0.99 (0.83-1.18)	.94	0.96 (0.80-1.15)	.64	0.95 (0.79-1.15)	.62
Donor related to recipient, non–cystic kidney disease^c^	0.95 (0.91-0.98)	.003	1.00 (0.96-1.05)	.99	1.08 (1.03-1.13)	.002	1.06 (1.01-1.12)	.01
Donor related to recipient, donor African American^d^	1.11 (0.99-1.24)	.09	1.13 (0.99-1.29)	.07	0.99 (0.86-1.14)	.91	0.98 (0.85-1.12)	.74
Donor related to recipient, donor not African American^e^	0.99 (0.95-1.03)	.55	1.05 (1.00-1.10)	.05	1.05 (1.00-1.10)	.06	1.04 (0.99-1.09)	.16

Abbreviation: HR, hazard ratio.

^a^ The HRs are for biologically related donor-recipient pair (reference group, unrelated donor-recipient pair). Model 1 variables: donor-recipient relationship, number of HLA-A mismatches, number of HLA-B mismatches, and number of HLA-DR mismatches. Model 2: model 1 plus donor age, donor sex, donor race (categorical variable; reference: White; groups: Black/African American, Hispanic, other; not included in models stratified by donor race), donor predonation creatinine level, recipient age (years), recipient sex, recipient diabetes, recipient peripheral vascular disease, recipient functional status (impaired vs unimpaired), recipient dialysis time (categorical variable; reference: preemptive transplant; groups: <1 year and ≥1 year, unknown), recipient insurance type (categorical variable; reference: private insurance; groups: Medicare, Medicaid, other), recipient employment status (reference: employed; categories: not employed, unknown employment status), recipient cause of kidney disease (cystic vs noncystic; not included in models stratified by cystic disease), and ABO-incompatible transplant. Model 3: model 2 plus era (2000-2004, 2005-2009, or 2010-2014, treated as a categorical variable).

^b^ Group sizes: related, n = 2530; not related, n = 4600.

^c^ Group sizes: related, n = 40 644; not related, n = 25 206.

^d^ Group sizes: related, n = 6958; not related, n = 2541.

^e^ Group sizes: related, n = 36 216; not related, n = 27 265.

Given the high frequency of known monogenic causes of cystic kidney disease, the cohort was stratified by recipients’ primary kidney disease. Although the association between donor-recipient biological relationship and death-censored allograft failure persisted in fully adjusted models when recipients had noncystic kidney disease (adjusted HR, 1.05; 95% CI, 1.01-1.10; *P* = .03), this association was not present among recipients with cystic kidney disease (adjusted HR, 1.03; 95% CI, 0.85-1.24; *P* = .77) (Table 3).

Finally, given the potential contribution of *APOL1* (OMIM 603743) risk variants to our findings,^14,15^ the cohort was stratified by donor race. In fully adjusted models, donor-recipient biological relationship was associated with increased death-censored allograft failure in transplants from African American donors (adjusted HR, 1.12; 95% CI, 1.01-1.23; *P* = .03) but not transplants from non–African American donors (adjusted HR, 1.03; 95% CI, 0.98-1.08; *P* = .20) (Table 3).

Overall results were similar using composite allograft failure as the outcome of interest, with the exception of the persistence of an association between donor-recipient biological relationship and composite allograft failure in adjusted models regardless of donor race (Table 3). Results were also similar using recipient death as an outcome, with donor-recipient biological relationship associated with increased death after transplant in adjusted models (adjusted HR, 1.06; 95% CI, 1.01-1.11; *P* = .02) (Table 3).

Short-term rejection within 1 year of transplant occurred in 6092 (8%) of transplants, including 2925 (10%) from unrelated donors and 3167 (7%) from related donors (eTable 1 in the Supplement). However, 15 894 (22%) of transplants were missing data about 1-year rejection (not related, 6247 [21%]; related, 9647 [22%]). Although donor-recipient biological relationship was associated with lower odds of 1-year rejection in unadjusted analyses limited to those without missing data (odds ratio, 0.74; 95% CI, 0.70-0.78), this association was not significant in adjusted models (model 1: OR, 1.04; 95% CI, 0.97-1.11; model 2: OR, 0.97; 95% CI, 0.90-1.04; model 3: OR, 0.94; 95% CI, 0.88-1.01) (eTable 2 in the Supplement).

Reported causes of allograft failure were significantly different among recipients who were related vs unrelated to their donors (eTable 3 in the Supplement). Approximately half of the allograft failures in each group were attributed to acute or chronic rejection (2647 [51%] in the not related group and 4197 [51%] in the related group), whereas fewer were attributed to recurrent disease (409 [8%] in the not related group vs 791 [10%] in the related group). However, these comparisons were limited by incomplete data because cause of allograft failure was listed as “other” or missing in 1455 (28%) of unrelated transplants and 2212 (27%) of related transplants.

Results of time-to-event analyses were similar after including more recent transplants in the study cohort (eFigure 6 and eTables 4, 5, and 6 in the Supplement).

## Discussion

This cohort study examined the association between donor-recipient biological relationship and posttransplant outcomes and found that, although both groups of allografts displayed similar unadjusted death-censored survival, allografts from living unrelated transplants had longer survival after HLA matching was taken into account. This association persisted after accounting for donor characteristics, recipient characteristics and transplant era, underscoring the need to determine the biological and sociodemographic factors underlying the differences. As the living donor pool shifts over time, it is crucial to understand the association of these changes with donor and recipient outcomes. In 2019, almost two-thirds of living kidney donors in the US were biologically unrelated to their recipient, the highest proportion ever observed.^1^ Given evidence that efforts to expand the living donor pool primarily increase living donor transplants from unrelated donors, this trend is likely to continue.^16,17^

One possibility for the association is that unrecognized shared genetic predispositions to kidney disease between recipients and biologically related donors contribute to the observed inferior outcomes. This hypothesis is consistent with prior evidence that donors who are biologically related to their recipients are more likely to develop end-stage kidney disease compared with their counterparts who are unrelated to their recipients.^11,12^ This issue has been a long-standing concern for recipients with cystic disease. As a result, related donor candidates are screened clinically (eg, with kidney imaging in autosomal dominant polycystic kidney disease) or more recently with genetic testing for known monogenic variants associated with cystic disease.^18^ As a result, related donor candidates with potential evidence of disease, those who are unable to be adequately screened, or those for whom the intended recipient has a genetic variant that has yet to be identified are likely to be excluded, potentially accounting for the absence of an increased risk of allograft failure from related donors in patients with cystic kidney disease in this study.

Complex polygenic predispositions to kidney failure, on the other hand, are not as readily identifiable, and many are yet to be recognized. In addition, the presence of known risk factors for kidney disease, such as *APOL1* risk variants, can also be challenging during donor evaluations, given that most individuals and allografts with these genetic variants will not experience kidney disease or allograft failure, respectively. The adjusted models in this study revealed that donor-recipient biological relationship was predominantly associated with allograft failure in transplants from African American donors, a finding perhaps attributable to a higher proportion of related donors with *APOL1* risk variants associated with shorter allograft survival and lower postdonation kidney function.^14,15,19,20^ The results of this study also suggest a greater association of donor-recipient biological relationship with allograft failure in mismatched pairs. This finding may reflect the fact that the small proportion of unrelated pairs with no HLA mismatches have a genetic distance that is more similar to related pairs than other unrelated pairs. Given that the observed frequency of 0 HLA mismatches is higher among unrelated pairs than expected by random chance from the general population, additional similarities not captured in this study are likely between these donors and recipients.

Alternatively, it is possible that the shared predisposition to kidney disease among related donors and recipients with kidney disease is not exclusively genetic but also associated with environmental factors, including socioeconomic status, that are shared by related individuals. Shared environmental exposures may be distant and hard to identify, such as childhood nutritional deficiencies, access to care, allostatic load, or environmental toxins.^21^ Although it is theoretically possible that recipients of allografts from close relatives would behave differently than recipients of allografts from unrelated donors resulting in differences in medication adherence after transplant, there is no evidence to support this notion. Furthermore, this hypothesis is contradicted by evidence that medication nonadherence after kidney transplant is predominantly driven by practical limitations (eg, forgetfulness), health literacy, and medication beliefs—factors likely not expected to be influenced by the donor-recipient relationship.^22,23,24,25^ Economic factors also play a large role in access to and outcomes after transplant.^26,27^ Although we included recipient insurance type and employment status in adjusted models, these surrogates may not adequately capture socioeconomic status, especially given the small proportion of recipients using Medicaid as primary payer.

This study did not find a significant association between donor-recipient biological relationship and 1-year rejection in adjusted models. Similarly, small differences in causes of allograft failure among transplant failures were of unclear clinical significance despite achieving statistical significance. However, both of these analyses were limited by incomplete or missing data. Improving understanding of the differences in outcomes for related vs unrelated transplants requires improved data capture of these elements.

Previous investigators have also studied the association between donor and recipient biological relationship and allograft outcomes. Terasaki et al^2^ examined 3-year allograft survival rates for kidney transplants from parental donors, spousal donors, and other unrelated donors and found similar allograft survival between groups despite differences in HLA matching. However, their study was limited by short follow-up and did not adjust for HLA mismatching when analyzing outcomes. Another study,^4^ of 1000 living donor transplants from 1966 to 1994, found no difference between haploidentical living unrelated and living related transplants. However, this single-center analysis was limited by the small proportion (9%) of donors unrelated to their recipients and was performed in a different era. Multiple investigators have demonstrated increased rates of acute rejection after living unrelated kidney transplants compared with living related kidney transplants, presumably because of higher degrees of HLA mismatching.^6,8^ Our results extend these findings to conclude that, although related and unrelated donor-recipient pairs have similar outcomes in unadjusted analysis, conditional on all else (including HLA matching), living unrelated kidney transplants appear to experience longer allograft survival.

Ultimately, delineating the causes of the association between donor-recipient biological relationship and allograft failure is key to understanding the implications of this finding. For example, it would be inappropriate to infer that paired kidney exchanges that involve 2 related donor-recipient pairs should be pursued simply for the sake of producing unrelated donor-recipient pairs because both donors would still be related to recipients with kidney failure. In addition, it still seems that optimizing HLA matching should be prioritized.^9^ For now, on the basis of this study’s findings, if choosing between an unrelated and related donor with similar degrees of HLA matching, it may be reasonable to choose an unrelated donor with no family history of kidney disease. Given the modest effect size of the study, the larger imperative is understanding the mechanism of this association. Broader investigational genetic testing of donors and recipients with extended follow-up is warranted to investigate the possibility of shared genetic predispositions to kidney disease as a mechanism underlying these findings. Although long-term biobanking of donor and recipient serum samples to allow future testing for as-yet-undiscovered risk variants should also be considered, these findings underscore the need for living donor programs to not overlook existing opportunities to identify genetic risk.^28,29^

### Strengths and Limitations

This study has strengths and limitations. Strengths include the use of a large, nationally representative cohort and clearly identified biological relationships between the donor and recipient. Limitations include the possibility of residual confounding because of donor or recipient characteristics not included here. Although dialysis exposure and preemptive status at transplant were similar between groups, unmeasured differences in the timing of transplant for each group could have confounded the results, especially given missing data regarding pretransplant dialysis time. Additional donor characteristics that may be relevant had a high degree of missingness in the data set. For example, the Living Donor Kidney Donor Profile Index includes several donor variables absent from the study models, including systolic blood pressure, smoking, and body mass index or weight.^30^ However, these variables could not be incorporated, given that 38% of donors were missing data on at least one. A high degree of concordance between donor and recipient race in the study cohort also precludes the ability to differentiate independent associations of each of these factors with posttransplant outcomes. Information about family history of kidney disease for living kidney donors was lacking, which may be more informative than donor-recipient biological relationship. Finally, the study cohort is only composed of transplants occurring in the US, and the results therefore may not be generalizable to other countries given known differences in outcomes for US vs other kidney transplant recipients.^31^

## Conclusions

Living donor kidney transplants from unrelated donors had longer allograft survival than those from related donors after HLA mismatching and donor and recipient characteristics were accounted for. Combined with prior evidence of an increased risk of kidney failure among donors related to their recipients, these findings should lead to additional study of the genetic and socioeconomic factors underlying familial clustering of kidney disease. These findings underscore the need for more precise risk assessments of kidney disease to guide living kidney donor screening and selection.

## Notes

Supplement. **eMethods.** Supplemental Methods **eTable 1.** Proportion of Living Donor Kidney Transplants in the Primary Analysis Experiencing Allograft Rejection in the First Post-Transplant Year, by Donor Type **eTable 2.** Association Between Donor-Recipient Biological Relationship and One-Year Allograft Rejection After Living Donor Kidney Transplant Among Recipients in the Primary Analysis Without Missing Data on Rejection (N = 57,086) **eTable 3.** Causes of Allograft Failure Among Living Donor Kidney Transplants in the Primary Analysis Experiencing Allograft Failure, by Donor Type **eTable 4.** Baseline Characteristics of Living Donor Kidney Transplant Donors and Recipients Included in the Secondary Analysis (2000-2019) **eTable 5.** Number of HLA Mismatches in Biologically Related Versus Unrelated Living Kidney Donor-Recipient Pairs in the Secondary Analysis (2000-2019) **eTable 6.** Association Between Donor-Recipient Biological Relationship and Allograft Failure After Living Donor Kidney Transplant in the Secondary Analysis (2000-2019) **eFigure 1.** Flow Diagram of Study Cohort for the Primary Analysis (2000-2014) **eFigure 2.** United States Living Donor Kidney Transplants Included in the Primary and Secondary Analysis, 2000-2019, by Donor Type **eFigure 3.** Unadjusted Kaplan-Meier Curves of Death-Censored Allograft Survival After Living Donor Kidney Transplant, 2000-2014, by the Total Number of Donor-Recipient HLA Mismatches (Log-Rank *P*<0.001 for Effect Across All Subgroups) **eFigure 4.** Unadjusted Kaplan-Meier Curves of Death-Censored Allograft Survival After Living Donor Kidney Transplant, 2000-2014, by Donor-Recipient Biological Relationship **eFigure 5.** Unadjusted Kaplan-Meier Curves of Death-Censored Allograft Survival After Living Donor Kidney Transplant, 2000-2014, by Donor-Recipient Biological Relationship, Stratified by the Total Number of Donor-Recipient HLA Mismatches **eFigure 6.** Flow Diagram of Study Cohort for Secondary Analysis (2000-2019)
