# Supplementary material for: Association Between Donor-Recipient Biological Relationship and Allograft Outcomes After Living Donor Kidney Transplant
Source: JAMA Netw Open. 2021 Apr 13;4(4):e215718. doi: 10.1001/jamanetworkopen.2021.5718 (PMC8044734; doi:10.1001/jamanetworkopen.2021.5718)
Supplement: Supplement. — eMethods. Supplemental Methods eTable 1. Proportion of Living Donor Kidney Transplants in the Primary Analysis Experiencing Allograft Rejection in the First Post-Transplant Year, by Donor Type eTable 2. Association Between Donor-Recipient Biological Relationship and One-Year Allograft Rejection After Living Donor Kidney Transplant Among Recipients in the Primary Analysis Without Missing Data on Rejection (N = 57,086) eTable 3. Causes of Allograft Failure Among Living Donor Kidney Transplants in the Primary Analysis Experiencing Allograft Failure, by Donor Type eTable 4. Baseline Characteristics of Living Donor Kidney Transplant Donors and Recipients Included in the Secondary Analysis (2000-2019) eTable 5. Number of HLA Mismatches in Biologically Related Versus Unrelated Living Kidney Donor-Recipient Pairs in the Secondary Analysis (2000-2019) eTable 6. Association Between Donor-Recipient Biological Relationship and Allograft Failure After Living Donor Kidney Transplant in the Secondary Analysis (2000-2019) eFigure 1. Flow Diagram of Study Cohort for the Primary Analysis (2000-2014) eFigure 2. United States Living Donor Kidney Transplants Included in the Primary and Secondary Analysis, 2000-2019, by Donor Type eFigure 3. Unadjusted Kaplan-Meier Curves of Death-Censored Allograft Survival After Living Donor Kidney Transplant, 2000-2014, by the Total Number of Donor-Recipient HLA Mismatches (Log-Rank P<0.001 for Effect Across All Subgroups) eFigure 4. Unadjusted Kaplan-Meier Curves of Death-Censored Allograft Survival After Living Donor Kidney Transplant, 2000-2014, by Donor-Recipient Biological Relationship eFigure 5. Unadjusted Kaplan-Meier Curves of Death-Censored Allograft Survival After Living Donor Kidney Transplant, 2000-2014, by Donor-Recipient Biological Relationship, Stratified by the Total Number of Donor-Recipient HLA Mismatches eFigure 6. Flow Diagram of Study Cohort for Secondary Analysis (2000-2019) [file jamanetwopen-e215718-s001.pdf]

Supplementary Online Content

Husain SA, King KL, Sanichar N, Crew RJ, Schold JD, Mohan S. Association between donor-recipient biological relationship and allograft outcomes after living donor kidney transplant. *JAMA Netw Open*. 2021;4(4):e215718. doi:10.1001/jamanetworkopen.2021.5718

eMethods. Supplemental Methods

eTable 1. Proportion of Living Donor Kidney Transplants in the Primary Analysis Experiencing Allograft Rejection in the First Post-Transplant Year, by Donor Type

eTable 2. Association Between Donor-Recipient Biological Relationship and One-Year Allograft Rejection After Living Donor Kidney Transplant Among Recipients in the Primary Analysis Without Missing Data on Rejection (N=57,086)

eTable 3. Causes of Allograft Failure Among Living Donor Kidney Transplants in the Primary Analysis Experiencing Allograft Failure, by Donor Type

eTable 4. Baseline Characteristics of Living Donor Kidney Transplant Donors and Recipients Included in the Secondary Analysis (2000-2019)

eTable 5. Number of HLA Mismatches in Biologically Related Versus Unrelated Living Kidney Donor-Recipient Pairs in the Secondary Analysis (2000-2019)

eTable 6. Association Between Donor-Recipient Biological Relationship and Allograft Failure After Living Donor Kidney Transplant in the Secondary Analysis (2000-2019)

eFigure 1. Flow Diagram of Study Cohort for the Primary Analysis (2000-2014)

eFigure 2. United States Living Donor Kidney Transplants Included in the Primary and Secondary Analysis, 2000-2019, by Donor Type

eFigure 3. Unadjusted Kaplan-Meier Curves of Death-Censored Allograft Survival After Living Donor Kidney Transplant, 2000-2014, by the Total Number of Donor-Recipient HLA Mismatches (Log-Rank *P*<0.001 for Effect Across All Subgroups)

eFigure 4. Unadjusted Kaplan-Meier Curves of Death-Censored Allograft Survival After Living Donor Kidney Transplant, 2000-2014, by Donor-Recipient Biological Relationship

eFigure 5. Unadjusted Kaplan-Meier Curves of Death-Censored Allograft Survival After Living Donor Kidney Transplant, 2000-2014, by Donor-Recipient Biological Relationship, Stratified by the Total Number of Donor-Recipient HLA Mismatches

eFigure 6. Flow Diagram of Study Cohort for Secondary Analysis (2000-2019)

This supplementary material has been provided by the authors to give readers additional information about their work.

© 2021 Husain SA et al. *JAMA Network Open.*

eMethods. Supplemental Methods

This was a retrospective cohort study using the Organ Procurement and Transplantation Network Standard Transplant Analysis and Research file (2020Q1). Recipient characteristics, donor characteristics, and transplant characteristics included in the analysis were selected *a priori* based on clinical importance/relevance and availability in this retrospective database.

Donor-recipient pairs were classified as related if any biological relationship was reported. Relationships classified as biological included donor as a parent (7%), child (19%), sibling (27%), or "Biological blood related- Other" (7%). Relationships classified as non-biological included spouse/life partner (13%), "Nonbiological other unrelated directed donation" (21%), and nonbiological unrelated paired/domino/anonymous donation (6%).

Adults were defined by age ≥18 years. Donor and recipient race were assigned as recorded in the dataset. In adjusted models, donor race was treated as a categorical variable (White, Black/African American, Hispanic, or Other) with White race as the reference group. Given concordance between donor and recipient race (>90% overall, including 95% concordance for Black/African American race and 92% concordance for White race) only donor race was included in adjusted models. Recipients were classified as having impaired functional status at transplant if functional status was recorded as requiring any assistance with activities of daily living, or if quantitated functional status was listed as having any greater than "minor" restrictions (i.e. if listed as having <90% functional status). Recipients were considered to have received a preemptive transplant if they did not receive dialysis prior to transplant. Recipient dialysis time was calculated as the difference between the date of transplant and the date of dialysis initiation. However, 21,586 recipients explicitly listed as receiving dialysis before transplantation had dates of dialysis initiation that were not included; in these cases, dialysis time was categorized as "Not preemptive but time not known." In adjusted models, dialysis time was treated as a categorical value, where pre-emptive transplantation (n=22,572) was the reference, compared to dialysis time <1 year (n=8,433), dialysis time ≥1 year (20,389), and unknown dialysis time (n=21,586). Recipients were classified as having high PRA if any reported PRA value (initial, peak, or last) was ≥80%. In all other cases, including those with all PRA values <80% and those with no PRA values recorded (25% overall, including 19% of recipients of unrelated transplants and 30% of recipients of related transplants), recipients were classified as not having high PRA. Recipient diabetes status was unknown or missing for 791 (0.8%) recipients; in these cases, the recipient was considered nondiabetic. Recipient employment status was classified as "employed" if the recipient was reported as working for income. Employment status was listed as unknown for 34% of recipients (28% of unrelated transplant recipients, 38% of related recipients). In multivariable analysis, employment status was treated as a categorical variable with 3 groups (employed, not employed, unknown) with "employed" as the reference group. Era was defined in 5-year increments (2000-2004, 2005-2009, or 2010-2014) and treated as a categorical variable, with 2000-2004 used as the reference group in multivariable analyses. A similar proportion of unrelated (3%) and related (4%) transplants were excluded due to missing data.

Recipients 1-year rejection status was classified as rejection, no rejection, or missing based on whether they were recorded as having rejection within the first year after transplant, regardless of the duration of follow up. Recorded causes of allograft failure were grouped into 6 categories: Acute or Chronic Rejection; Recurrent Disease; Surgical, Urologic, or Thrombosis; Infection (including polyomavirus); Primary Nonfunction; or Other. Cause of failure was only categorized as "Other" when it was recorded as "999- Other."

Time of inception for the cohorts was at the time of transplant. Participants lost to follow up were censored at last follow up. Total analysis time for the primary analysis was 572,215 years, and median time to allograft failure, censoring, or end of follow up was 7.5 years for related transplants and 7.0 years for unrelated transplants. Visual inspection of -ln[ln(survival)] plots for donor-recipient biological relationship were used to confirm that Cox models satisfied the proportional hazards assumption.

© 2021 Husain SA et al. *JAMA Network Open.*

eTable 1. Proportion of living donor kidney transplants in the primary analysis experiencing allograft rejection in the first post-transplant year, by donor type.

	**Not Related**	**Related**
n (column %)	**29,806 (41%)**	**43,174 (59%)**
No 1-year rejection	20634 (69)	30360 (70)
1-year rejection	2925 (10)	3167 (7)
Missing data	6247 (21)	9647 (22)

p<0.001 for table-wide comparison

© 2021 Husain SA et al. *JAMA Network Open.*

eTable 2. Association between donor-recipient biological relationship and one-year allograft rejection after living donor kidney transplant among recipients in the primary analysis without missing data on rejection (n=57,086)

	**Unadjusted**		**Model 1**		**Model 2**		**Model 3**	
	**OR (95% CI)**	**p**	**OR (95% CI)**	**p**	**OR (95% CI)**	**p**	**OR (95% CI)**	**p**
Donor related to recipient	0.74 (0.70-0.78)	<0.001	1.04 (0.97-1.11)	0.27	0.97 (0.90-1.04)	0.34	0.94 (0.88-1.01)	0.09

*Odds ratios are is for biologically related donor-recipient pair (reference group = unrelated donor-recipient pair)
Model 1 variables: Donor-recipient relationship, number of HLA A mismatches, number of HLA B mismatches, number of HLA DR mismatches
Model 2: Model 1 + donor age, donor sex, donor race (categorical variable; reference: White; groups: Black/African American, Hispanic, Other; not included in models stratified by donor race), donor pre-donation creatinine (mg/dL), recipient age (years), recipient sex, recipient diabetes, recipient peripheral vascular disease, recipient functional status (impaired versus unimpaired), recipient dialysis time (categorical variable; reference: pre-emptive transplant; groups: <1 year, ≥1 year, unknown), recipient insurance type (categorical variable; reference: private insurance; groups: Medicare, Medicaid, Other), recipient employment status (reference: employed; categories: not employed, unknown employment status), recipient cause of kidney disease (cystic vs non-cystic; not included in models stratified by cystic disease), and ABO-incompatible transplant
Model 3: Model 2 + era (2000-2004, 2005-2009, 2010-2014, or 2015-2019, treated as a categorical variable)

© 2021 Husain SA et al. *JAMA Network Open.*

eTable 3. Causes of allograft failure among living donor kidney transplants in the primary analysis experiencing allograft failure, by donor type.

	**Not Related**	**Related**
n (column %)	**n=5214, 39%**	**n=8155, 61%**
Acute or Chronic Rejection	2647 (51)	4197 (51)
Recurrent Disease	409 (8)	791 (10)
Surgical, Urologic, or Thrombosis	240 (5)	379 (5)
Infection (including polyomavirus)	287 (6)	297 (4)
Primary Nonfunction	176 (3)	279 (3)
Other	1426 (27)	2176 (27)
Missing	29 (0.6)	36 (0.4)

$p<0.001$ for table-wide comparison

© 2021 Husain SA et al. *JAMA Network Open.*

eTable 4. Baseline characteristics of living donor kidney transplant donors and recipients included in the secondary analysis (2000-2019).

	All	Not Related	Related	
n (column %) or median (IQR)	**98,419 (100%)**	**44,553 (45%)**	**53,866 (55%)**	**p**
**Donor Characteristics**				
Age, years	42 (33-51)	45 (36-53)	40 (31-49)	<0.001
Female	60,211 (61%)	28,901 (65%)	31,310 (58%)	<0.001
Race				<0.001
White	67,713 (69%)	34,417 (77%)	33,296 (62%)	
Black/African American	11,868 (12%)	3,531 (8%)	8,337 (15%)	
Hispanic/Latino	13,604 (14%)	4,629 (10%)	8,975 (17%)	
Pre-donation serum creatinine (mg/dL)	0.80 (0.70 - 1.00)	0.80 (0.70 - 0.96)	0.83 (0.70 - 1.00)	<0.001
**Recipient Characteristics**				
Age, years	49 (38-59)	50 (41-59)	48 (35-59)	<0.001
Female	37,878 (38%)	15,819 (36%)	22,059 (41%)	<0.001
Race				<0.001
White	64,258 (65%)	20,983 (70%)	33,275 (62%)	
Black/African American	14,044 (14%)	5,619 (13%)	8,425 (16%)	
Hispanic/Latino	13,927 (14%)	5,051 (11%)	8,876 (16%)	
Cause of kidney disease				<0.001
Diabetes	19,294 (20%)	9,335 (21%)	9,959 (18%)	
Hypertension	14,288 (15%)	6,311 (14%)	7,977 (15%)	
Glomerular disease	23,449 (24%)	10,488 (24%)	12,961 (24%)	
Cystic kidney disease	10,767 (11%)	7,270 (16%)	3,497 (6%)	
Other/Unknown	30,621 (31%)	11,149 (25%)	19,472 (36%)	
Panel reactive antibody ≥ 80%	2,234 (2%)	1,078 (2%)	1,156 (2%)	0.004
Diabetes	28,365 (29%)	13,014 (29%)	15,351 (29%)	0.01
Peripheral vascular disease	5,120 (5%)	2,471 (6%)	2,649 (5%)	<0.001
Functional impairment	57,928 (59%)	27,874 (63%)	30,054 (56%)	<0.001
Pre-transplant dialysis				
Pre-emptive (no dialysis)	31,625 (32%)	14,821 (33%)	16,804 (31%)	<0.001
<1 year	12,358 (13%)	5,252 (12%)	7,106 (13%)	
1 year or more	29,810 (30%)	14,408 (32%)	15,402 (29%)	
Not preemptive but time not known	24,626 (25%)	10,072 (23%)	14,554 (27%)	
Employment status				
Employed	35,532 (36%)	18,688 (42%)	16,844 (31%)	<0.001
Not employed	37,121 (38%)	16,955 (38%)	20,166 (37%)	
Unknown/missing	25,766 (26%)	8,910 (20%)	16,856 (31%)	
Insurance				
Private	56,990 (58%)	26,951 (60%)	30,039 (56%)	<0.001
Medicare	29,095 (30%)	13,595 (31%)	15,500 (29%)	
Medicaid	3,939 (4%)	1,320 (3%)	2,619 (5%)	
Other	8,395 (9%)	2,687 (6%)	5,708 (11%)	

© 2021 Husain SA et al. *JAMA Network Open.*

**Other Characteristics**				
ABO incompatible transplant	1,248 (1%)	700 (2%)	548 (1%)	<0.001
Transplant Era				
2000-2004	23,655 (24%)	7,577 (17%)	16,078 (30%)	<0.001
2005-2009	25,460 (26%)	10,342 (23%)	15,118 (28%)	
2010-2014	23,865 (24%)	11,887 (27%)	11,978 (22%)	
2015-2019	25,439 (26%)	14,747 (33%)	10,692 (20%)	

© 2021 Husain SA et al. *JAMA Network Open.*

eTable 5. Number of HLA mismatches in biologically related versus unrelated living kidney donor-recipient pairs in the secondary analysis (2000-2019).

	**All**	**Not Related**	**Related**	
column %	**98,419 (100%)**	**44,553 (45%)**	**53,866 (55%)**	**p**
Total				
0	8%	0.4%	14%	<0.001
1	5%	0.9%	8%	
2	15%	4%	25%	
3	26%	14%	37%	
4	16%	27%	7%	
5	19%	34%	7%	
6	11%	19%	4%	
HLA A				
0	21%	9%	32%	<0.001
1	52%	44%	59%	
2	27%	47%	10%	
HLA B				
0	14%	3%	23%	<0.001
1	48%	30%	63%	
2	38%	67%	14%	
HLA DR				
0	19%	7%	29%	<0.001
1	52%	42%	60%	
2	29%	51%	11%	

© 2021 Husain SA et al. *JAMA Network Open.*

eTable 6. Association between donor-recipient biological relationship and allograft failure after living donor kidney transplant in the secondary analysis (2000-2019).

	**Unadjusted**		**Model 1**		**Model 2**		**Model 3**	
	**HR (95% CI)**	**p**	**HR (95% CI)**	**p**	**HR (95% CI)**	**p**	**HR (95% CI)**	**p**
**Death-Censored Graft Failure**								
Donor related to recipient- Full Cohort	1.02 (0.98-1.05)	0.36	1.26 (1.21-1.32)	<0.001	1.07 (1.02-1.11)	0.003	1.04 (1.00-1.09)	0.05
Donor related to recipient- Cystic Kidney Disease	0.91 (0.78-1.06)	0.21	1.08 (0.91-1.29)	0.37	1.04 (0.87-1.24)	0.69	1.02 (0.85-1.22)	0.84
Donor related to recipient- Non-cystic Kidney Disease	0.96 (0.93-0.99)	0.02	1.20 (1.15-1.25)	<0.001	1.07 (1.02-1.11)	0.003	1.05 (1.00-1.09)	0.04
Donor related to recipient- Donor African American	1.03 (0.95-1.12)	0.44	1.18 (1.07-1.30)	0.001	1.12 (1.02-1.24)	0.02	1.10 (1.00-1.21)	0.05
Donor related to recipient- Donor not African American	0.94 (0.91-0.98)	0.002	1.16 (1.11-1.21)	<0.001	1.05 (1.00-1.10)	0.05	1.03 (0.98-1.07)	0.27
**Composite Graft Failure**								
Donor related to recipient- Full Cohort	1.02 (1.00-1.05)	0.11	1.16 (1.13-1.20)	<0.001	1.13 (1.09-1.17)	<0.001	1.11 (1.07-1.15)	<0.001
Donor related to recipient- Cystic Kidney Disease	0.95 (0.85-1.06)	0.34	1.05 (0.92-1.19)	0.48	1.06 (0.93-1.20)	0.41	1.04 (0.92-1.19)	0.51
Donor related to recipient- Non-cystic Kidney Disease	0.97 (0.94-0.99)	0.01	1.11 (1.07-1.44)	<0.001	1.13 (1.10-1.17)	<0.001	1.11 (1.08-1.15)	<0.001
Donor related to recipient- Donor African American	1.05 (0.98-1.13)	0.17	1.16 (1.07-1.25)	<0.001	1.12 (1.03-1.21)	0.01	1.09 (1.01-1.19)	0.04
Donor related to recipient- Donor not African American	0.98 (0.96-1.01)	0.19	1.11 (1.07-1.15)	<0.001	1.11 (1.08-1.15)	<0.001	1.09 (1.06-1.13)	<0.001
**Recipient Death**								
Donor related to recipient- Full Cohort	1.00 (0.97-1.03)	0.95	1.05 (1.00-1.09)	0.03	1.07 (1.03-1.12)	0.002	1.05 (1.01-1.10)	0.02
Donor related to recipient- Cystic Kidney Disease	0.94 (0.82-1.08)	0.40	0.97 (0.82-1.15)	0.74	0.94 (0.79-1.13)	0.51	0.94 (0.78-1.12)	0.48
Donor related to recipient- Non-cystic Kidney Disease	0.95 (0.92-0.98)	0.003	1.00 (0.96-1.04)	0.95	1.08 (1.03-1.13)	0.001	1.06 (1.01-1.11)	0.01
Donor related to recipient- Donor African American	1.10 (0.98-1.23)	0.10	1.12 (0.98-1.27)	0.10	0.98 (0.85-1.12)	0.80	0.96 (0.83-1.10)	0.54

© 2021 Husain SA et al. *JAMA Network Open.*

Donor related to recipient- Donor not African American	0.99 (0.96-1.03)	0.63	1.05 (1.00-1.09)	0.05	1.05 (1.00-1.10)	0.05	1.03 (0.98-1.08)	0.21

Hazard ratios are for biologically related donor-recipient pair (reference group = unrelated donor-recipient pair)
Model 1 variables: Donor-recipient relationship, number of HLA-A mismatches, number of HLA-B mismatches, number of HLA-DR mismatches
Model 2: Model 1 + donor age, donor sex, donor race (categorical variable; reference: White; groups: Black/African American, Hispanic, Other; not included in models stratified by donor race), donor pre-donation creatinine (mg/dL), recipient age (years), recipient sex, recipient diabetes, recipient peripheral vascular disease, recipient functional status (impaired versus unimpaired), recipient dialysis time (categorical variable; reference: pre-emptive transplant; groups: <1 year, ≥1 year, unknown), recipient insurance type (categorical variable; reference: private insurance; groups: Medicare, Medicaid, Other), recipient employment status (reference: employed; categories: not employed, unknown employment status), recipient cause of kidney disease (cystic vs non-cystic; not included in models stratified by cystic disease), and ABO-incompatible transplant
Model 3: Model 2 + era (2000-2004, 2005-2009, 2010-2014, or 2015-2019, treated as a categorical variable)

© 2021 Husain SA et al. *JAMA Network Open.*

eFigure 1. Flow diagram of the study cohort for the primary analysis (2000-2014).

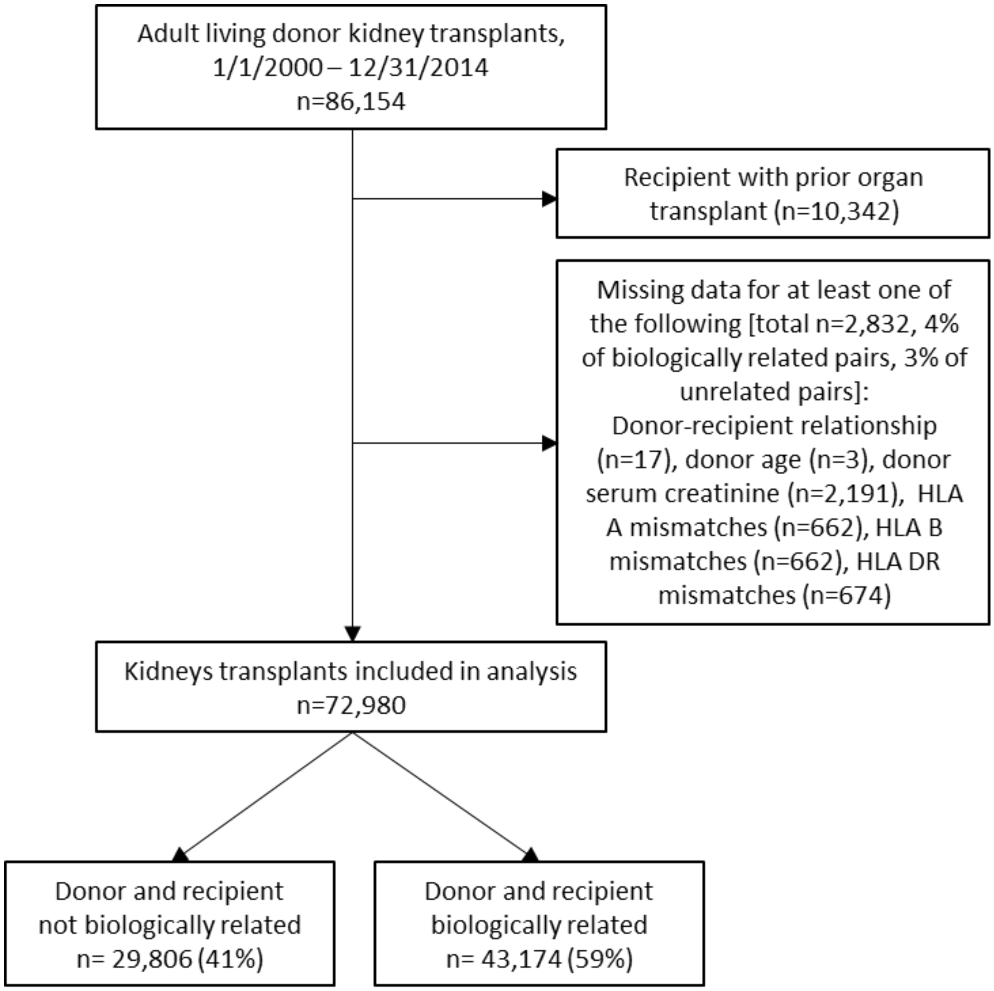

© 2021 Husain SA et al. *JAMA Network Open.*

eFigure 2. United States living donor kidney transplants included in the primary and secondary analysis, 2000-2019, by donor type. Each bar represents the total number of living donor transplants during a given 5-year era, with blue sections showing the number of donor-recipient pairs without a biological relationship, and grey sections showing the number of biologically related donor-recipient pairs. Although the total number of transplants was similar in each era, the proportion of transplants from donors with no biological relationship to their recipient increased from 32% (2000-2004) to 58% (2015-2019) during the study period.

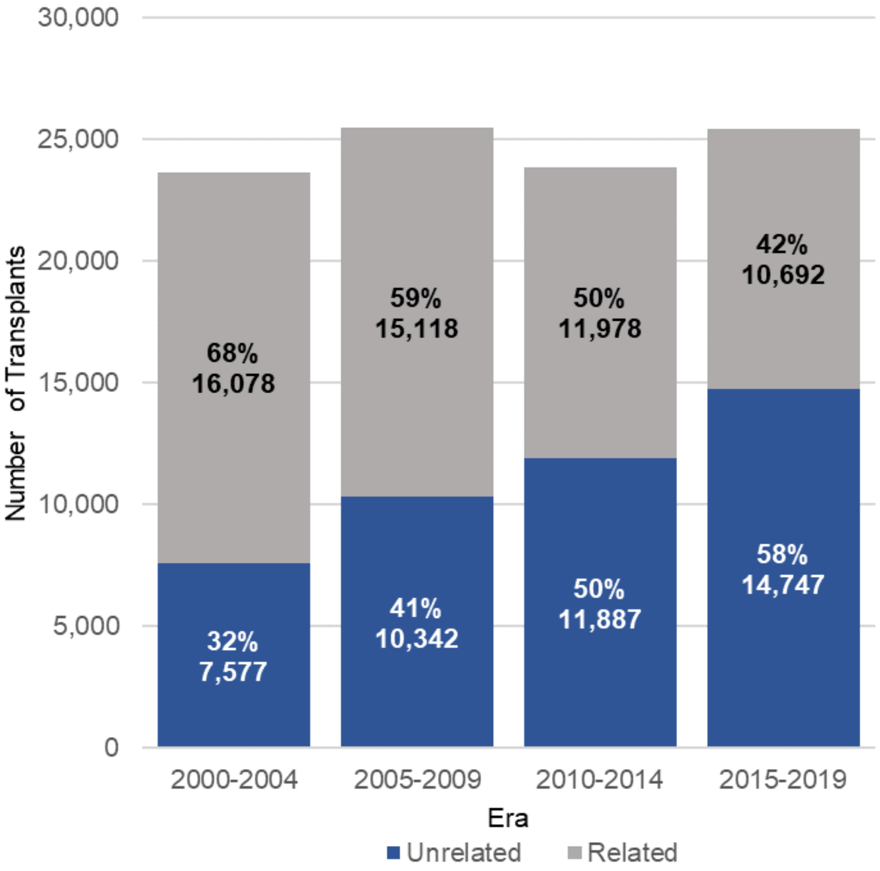

© 2021 Husain SA et al. *JAMA Network Open.*

eFigure 3. Unadjusted Kaplan-Meier curves of death-censored allograft survival after living donor kidney transplant, 2000-2014 ,by the total number of donor-recipient HLA mismatches (log-rank p<0.001 for effect across all subgroups).

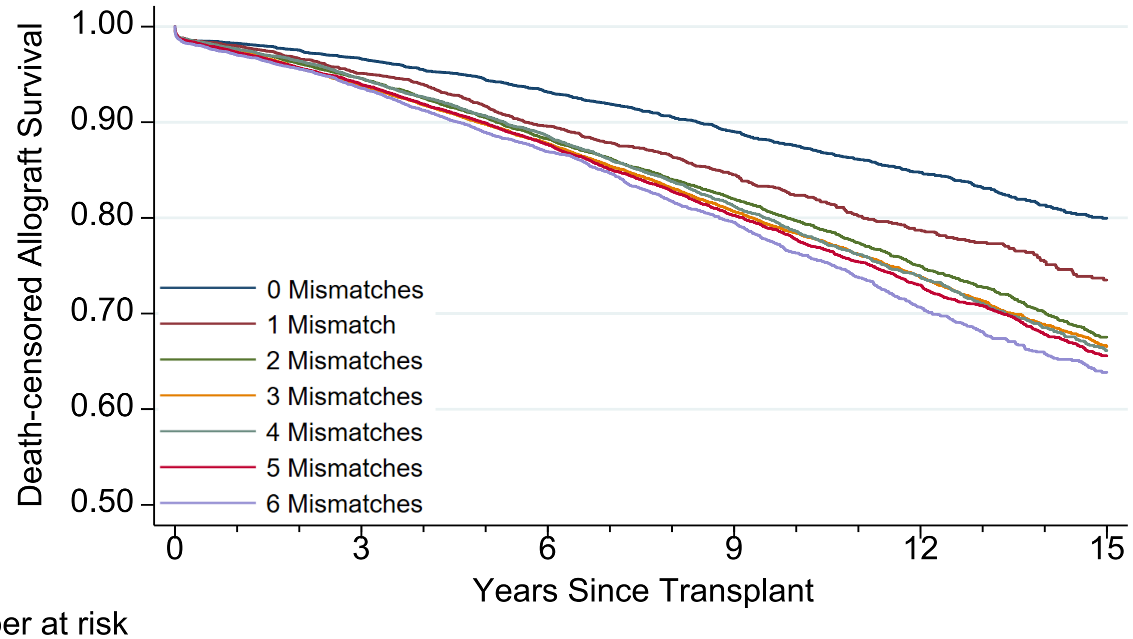

Number at risk

0 Mismatches	6064	5275	4033	2671	1566	717
1 Mismatch	3824	3259	2369	1479	826	331
2 Mismatches	11821	10070	7329	4566	2402	976
3 Mismatches	19928	16738	12111	7278	3809	1488
4 Mismatches	11025	9442	6703	3891	1976	753
5 Mismatches	13030	11074	7818	4389	2174	870
6 Mismatches	7288	6181	4348	2439	1172	423

© 2021 Husain SA et al. *JAMA Network Open.*

eFigure 4. Unadjusted Kaplan-Meier curves of death-censored allograft survival after living donor kidney transplant, 2000-2014, by donor-recipient biological relationship. There was no association between donor-recipient relationship and death-censored allograft failure (log-rank p=0.76).

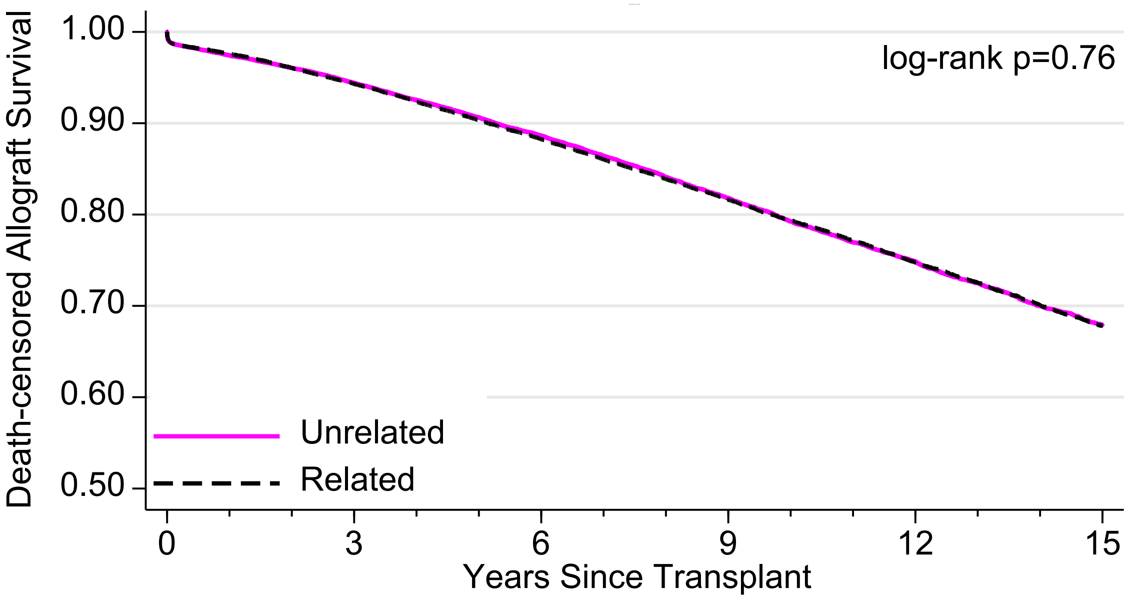

Number at risk

	0	3	6	9	12	15
Unrelated	29806	25505	17926	10021	4854	1784
Related	43174	36534	26785	16692	9071	3774

© 2021 Husain SA et al. *JAMA Network Open.*

eFigure 5. Unadjusted Kaplan-Meier curves of death-censored allograft survival after living donor kidney transplant, 2000-2014, by donor-recipient biological relationship, stratified by the total number of donor-recipient HLA mismatches.

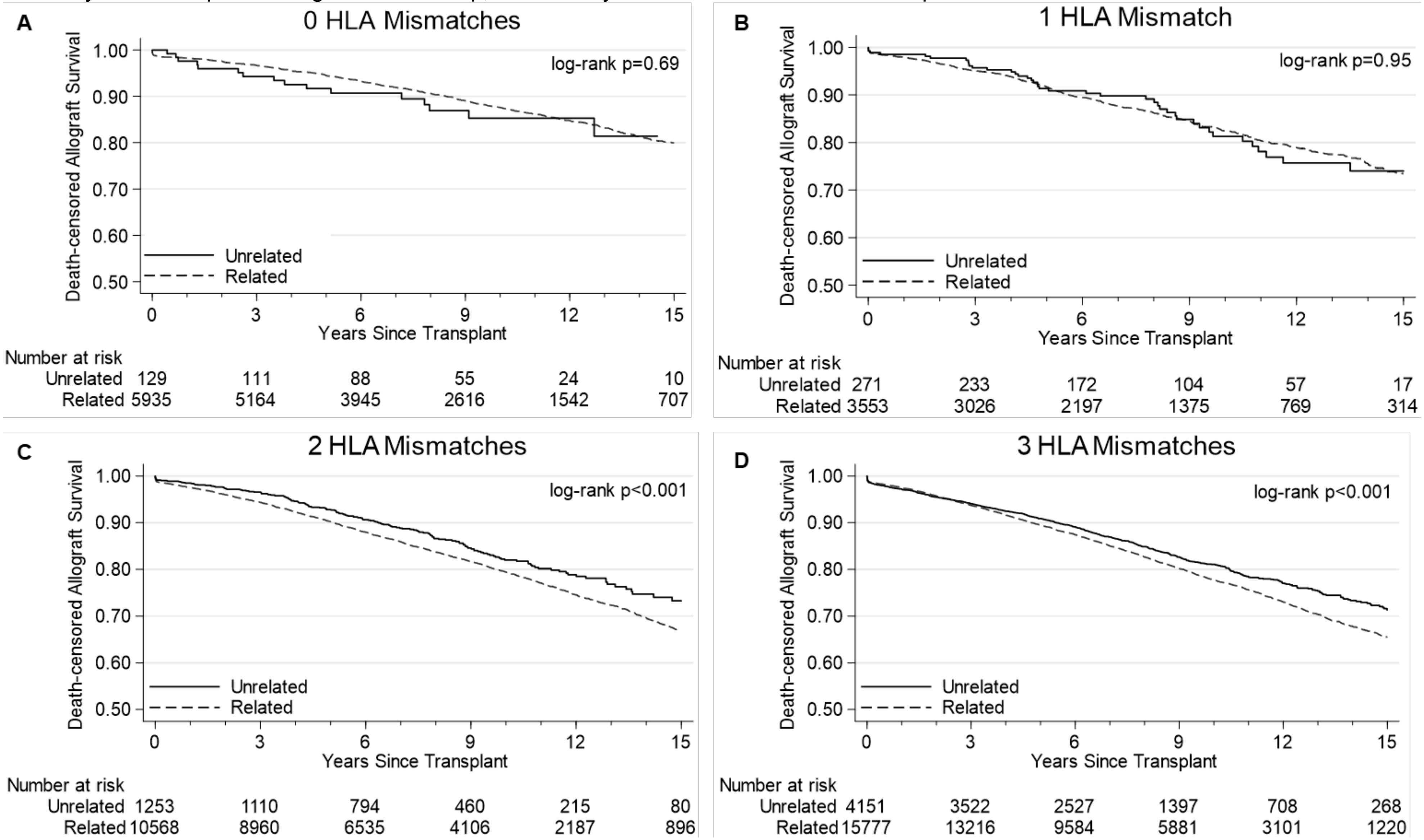

© 2021 Husain SA et al. *JAMA Network Open.*

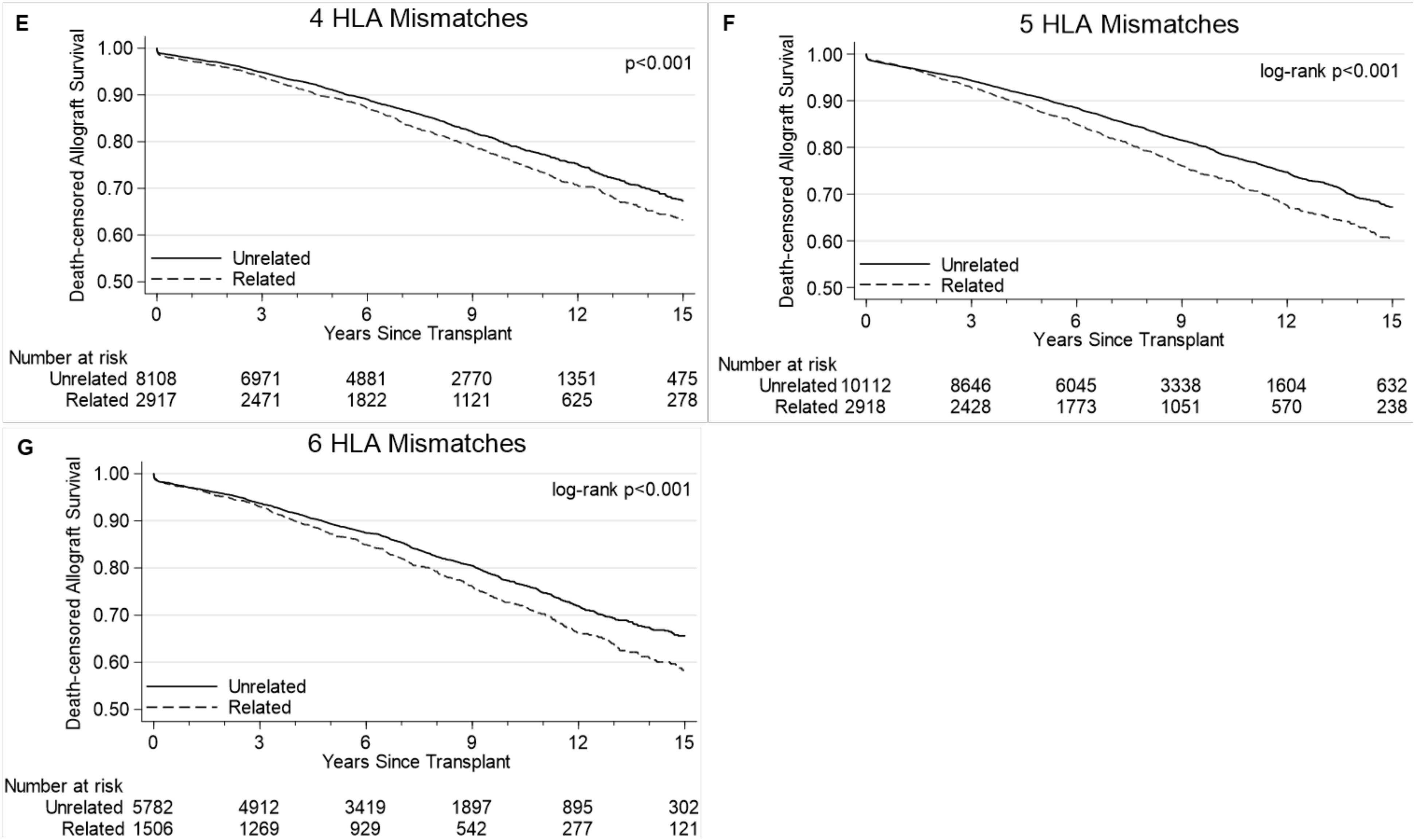

© 2021 Husain SA et al. *JAMA Network Open.*

eFigure 6. Flow diagram of the study cohort for the secondary analysis (2000-2019).

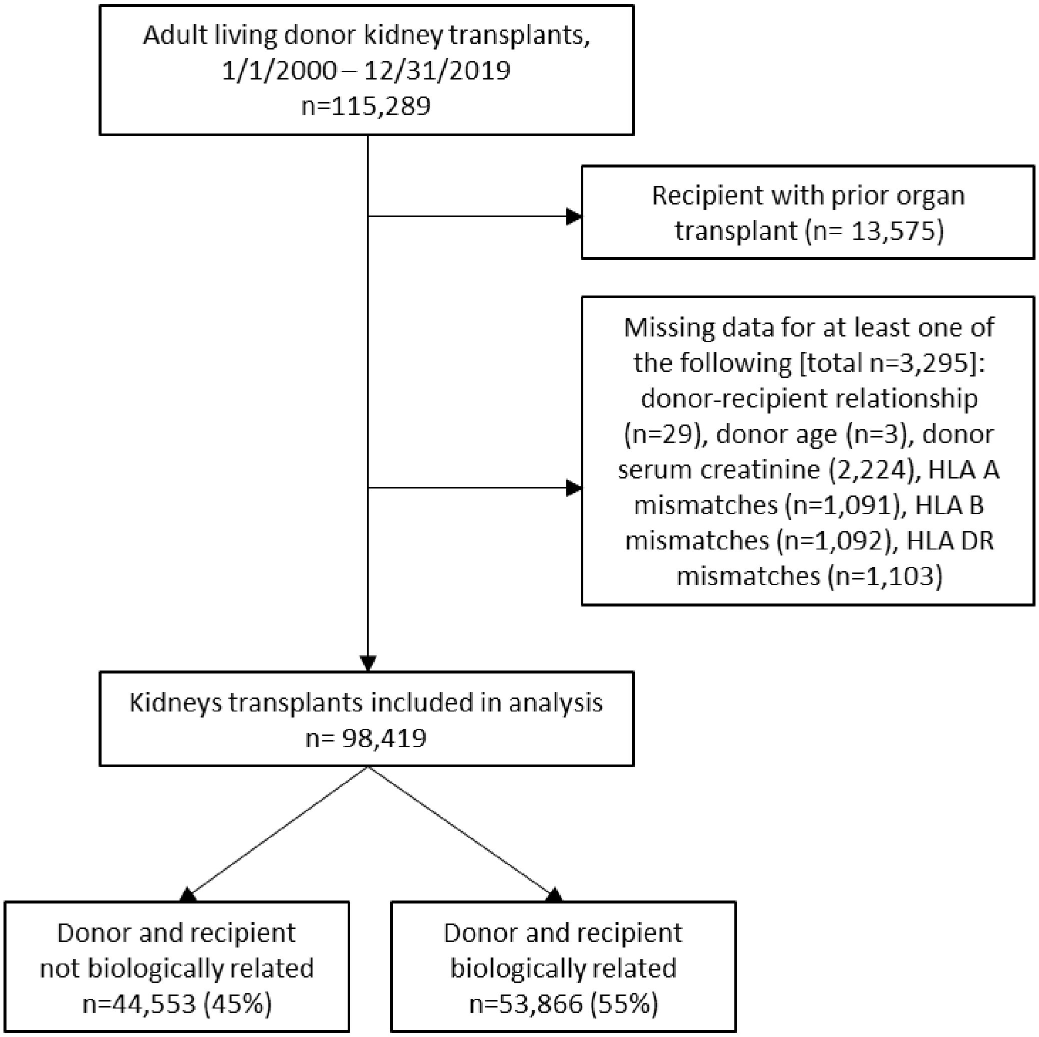

© 2021 Husain SA et al. *JAMA Network Open.*
